# Genomic Profiling of Circulating Tumor DNA Predicts Outcome and Demonstrates Tumor Evolution in ALK-Positive Non-Small Cell Lung Cancer Patients

**DOI:** 10.3390/cancers12040947

**Published:** 2020-04-11

**Authors:** Anne Tranberg Madsen, Anne Winther-Larsen, Tine McCulloch, Peter Meldgaard, Boe Sandahl Sorensen

**Affiliations:** 1 Department of Clinical Biochemistry, Aarhus University Hospital, 8200 Aarhus N, Denmark; Anne.Winther.Larsen@aarhus.rm.dk (A.W.-L.); boesoere@rm.dk (B.S.S.); 2 Department of Oncology, Aalborg University Hospital, 9000 Aalborg, Denmark; tine.mcculloch@rn.dk; 3 Department of Oncology, Aarhus University Hospital, 8200 Aarhus N, Denmark; petemeld@rm.dk

**Keywords:** serial monitoring, next-generation sequencing, ALK tyrosine kinase receptor, cell-free DNA, circulating tumor DNA, targeted therapy

## Abstract

With the rapid development of targeted therapies for the treatment of cancer, methods for predicting response and outcome are in high demand. Non-small cell lung cancer driven by genomic rearrangements of the anaplastic lymphoma kinase (*ALK*) gene can be successfully treated with ALK-targeted therapy. Unfortunately, a subset of patients does not respond, and all patients ultimately acquire resistance, highlighting the need for better clinical tools to manage these patients. Here, we performed targeted next-generation sequencing on plasma circulating tumor DNA (ctDNA) from 24 patients to assess the clinical utility of ctDNA genomic profiling. Patients with detectable ctDNA prior to treatment had worse progression-free survival (PFS) than those without (median 8.7 vs. 15.2 months, *p* = 0.028). In addition, the presence of ctDNA within two months after treatment initiation predicted inferior PFS (median 4.6 vs. 14.5 months, *p* = 0.028). Longitudinal monitoring of ctDNA with droplet digital PCR during treatment reflected the radiological response and revealed potential acquired resistance mutations. Interestingly, an increase in the ctDNA concentration was evident prior to the determination of progressive disease by conventional radiological imaging, with a median lead time of 69 days (range 30–113). Genomic profiling of ctDNA is a promising tool for predicting outcome and monitoring response to targeted therapy.

## 1. Introduction

Targeted therapies are becoming standard treatment for multiple cancer types, and several targeted therapy options are available for the treatment of non-small cell lung cancer (NSCLC). Rearrangements of the anaplastic lymphoma kinase (*ALK*) gene are found in a subset of NSCLC patients and drive tumor growth. Thus, the tumor cells are susceptible to targeted therapy using small tyrosine kinase inhibitors (TKIs). Currently, it is recommended that all NSCLC patients with an adenocarcinoma component are tested for *ALK* rearrangements in their diagnostic biopsy, and several ALK TKIs are approved for the treatment of ALK-positive patients [1,2]. Unfortunately, not all patients respond to the treatment, and all patients eventually acquire resistance and experience disease progression.

The mechanisms of resistance to ALK TKIs have been thoroughly investigated in the last few years [3,4]. A multitude of ALK kinase domain mutations can confer resistance, and each mutation imparts unique sensitivity characteristics to the various ALK TKIs [3,5,6]. Other resistance mechanisms include activation of bypass signaling pathways, such as the epidermal growth factor receptor (EGFR), while the mechanism remains unidentified for some patients [3,7]. Thus, genomic profiling of the tumor at the time of progression may help guide the selection of subsequent therapy. Unfortunately, the acquisition and application of tumor biopsies is not straightforward. Firstly, tumor biopsies are not always obtainable, and there is often inadequate material for multiple analyses [8,9,10]. In addition, they are spatially limited and may not reflect the inter- and intratumor molecular heterogeneity known to exist in lung cancer [11,12].

A noninvasive alternative to tumor biopsies is circulating tumor DNA (ctDNA). It comprises a small component of the total cell-free DNA (cfDNA), which can be found in plasma obtained from a blood sample. Analysis of ctDNA can provide genomic information on all tumor sites and sub-clones in a patient, and the ease of repeated sampling enables real-time longitudinal monitoring of the evolving genomic composition of the tumor [13,14,15,16,17].

In the present study, we performed ctDNA analysis using targeted next-generation sequencing (NGS) with the cancer personalized profiling by deep sequencing (CAPP-Seq) technology [18,19] on samples prior to and following ALK-TKI treatment to study the genomic composition of ALK-positive NSCLC in a real-world setting. Furthermore, we employed droplet digital PCR (ddPCR) to conduct longitudinal monitoring of select alterations during treatment with multiple lines of ALK TKIs. We show that upfront ctDNA analysis can predict treatment outcome and that longitudinal ctDNA analyses mirror clinical and radiological evaluations. Thus, genomic profiling using ctDNA could be a helpful non-invasive tool for the management of ALK-positive NSCLC patients.

## 2. Results

### 2.1. Patient Characteristics

**Figure 1:**
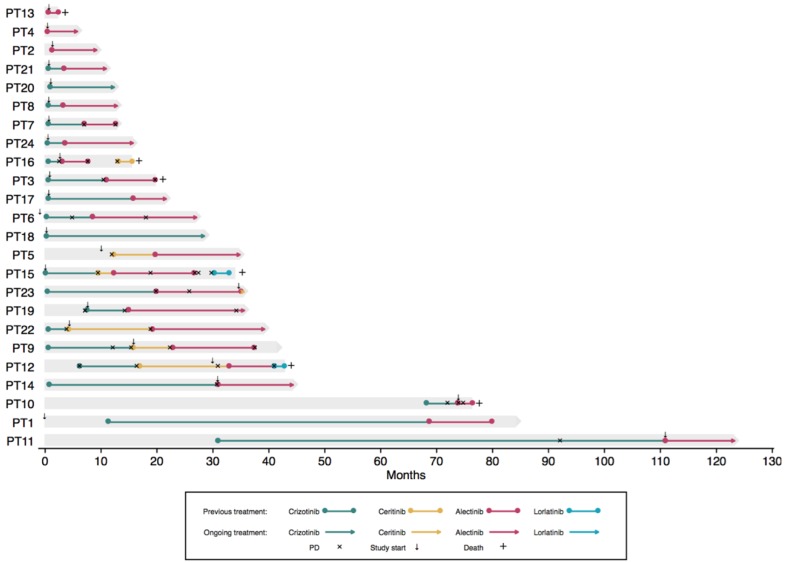
Individual treatment trajectories. The *x*-axis depicts months since diagnosis, and the *y*-axis depicts each patient. Deceased patients are marked by +, and progression is marked by ×. The small arrows mark when the patient was included in the present study. PD, progressive disease.

A total of 24 patients with advanced-stage ALK-positive NSCLC were included during the study period. All patients were diagnosed with an *ALK* rearrangement in their tumor biopsy. Patient characteristics are shown in Table 1 and Table S1. The majority of patients (19 of 24) had stage IV adenocarcinoma and was treatment naïve (14 of 24). The median follow-up time was 21 months (95% CI: 12–28). At the last follow-up date, 14 patients had experienced disease progression on at least one ALK TKI, and six patients were deceased. Treatment trajectories can be seen in Figure 1.

### 2.2. ctDNA Profiling by Targeted NGS

**Figure 2:**
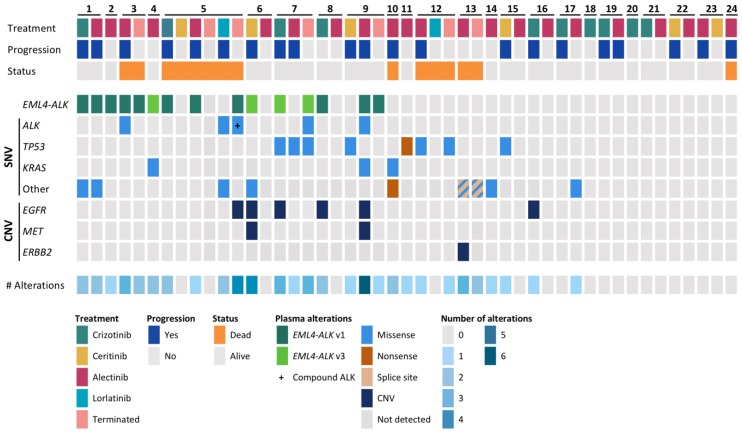
Overview of the basic characteristics and genomic alterations detected in circulating tumor DNA (ctDNA). The upper panel represents the treatment and outcome of the patients. The middle panel shows the alterations detected in ctDNA, pre-treatment or at treatment termination. The lower panel shows the number of alterations found in each sample. SNV, single-nucleotide variant; CNV, copy number variation; Terminated, sample taken at treatment termination due to progression or death.

To evaluate the genomic profile of the patients, cfDNA samples obtained before treatment start and at progression on any ALK TKI were subjected to targeted NGS with the AVENIO ctDNA Expanded Kit. A total of 47 cfDNA samples were analyzed, out of which 40 samples were pretreatment samples, and 7 were samples acquired at treatment termination. Genomic alterations were detected in 28/47 samples (60%) with a median single-nucleotide variant (SNV) allele frequency (AF) of 0.35% (range 0.10–79.5). The median number of alterations detected per sample was 1 (range 0–6). ALK rearrangements were found in 15 samples from 9 patients (9/24, 37.5%), and ALK mutations were identified in samples from four patients (p.C1156Y [PT3], p.G1202R [PT7], p.L1196M + p.G1202R [PT9], p.L11996M + p.D1203N [PT5]). Additionally, mutations were detected in TP53 in five patients (21%) and KRAS in three (13%) patients. Copy number variations (CNVs) of EGFR were identified in six patients, while CNVs of MET were identified in two patients, and CNVs of ERBB2 were found in one patient. In seven patients, no mutations were found in any samples (29%). An overview of alterations found by targeted NGS can be found in Figure 2 and Table S2.

### 2.3. Presence of ctDNA Alterations at Baseline Predicts Outcome

**Figure 3:**
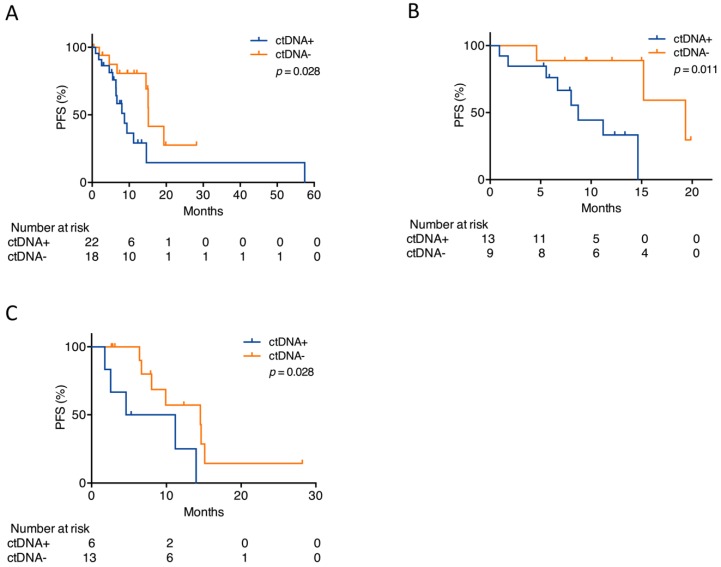
Detection of alterations before or early following ALK TKI start. (**A**) Kaplan–Meier plot for patients with detectable (ctDNA+) or undetectable (ctDNA−) alterations prior to start on any ALK TKI. (**B**) Kaplan–Meier plot for patients with detectable (ctDNA+) or undetectable (ctDNA−) alterations prior to alectinib start. (**C**) Kaplan–Meier plot for patients with detectable (ctDNA+) or undetectable (ctDNA−) alterations within the first two months on any ALK TKI. The *p*-values were determined by the log-rank test.

To investigate whether genomic profiling of cfDNA holds predictive power, the patients were dichotomized based on the presence of somatic alterations at initiation of the ALK TKI treatment. As some patients were treated with several ALK TKIs during the study period, and thus had more than one pretreatment sample, 40 pretreatment samples were available from the 24 patients. Alterations were detected in 22/40 (55%) pretreatment samples. When all pretreatment samples were combined into one analysis, disregarding the type of ALK TKI administered, patients with detectable ctDNA alterations prior to treatment had significantly shorter progression-free survival (PFS) (median 8.7 months (95% CI 5.3–12.1)) than patients with undetectable alterations (median 15.2 months (95% CI 15.0–15.4), *p* = 0.028) (Figure 3A). In a subgroup analysis, solely alectinib pretreatment samples (*n* = 22) were included in the analysis. CtDNA alterations were found in 13 of these samples, and the presence of ctDNA alterations was still significantly associated with an inferior PFS (8.7 months (95% CI 6.8–10.6) vs. 19.3 months (95% CI 12.6–26.0), *p* = 0.011) (Figure 3B).

### 2.4. Early Changes in ctDNA Alterations Correlate with Outcome

To examine whether the detection of alterations early after treatment initiation could predict outcome, a sample collected within two months after ALK TKI treatment start was subjected to targeted NGS (*n* = 19). The samples were collected a median of 28 days (range 8–56) after treatment initiation. Patients who had detectable ctDNA alterations in their first sample after treatment initiation had significantly shorter PFS (4.6 months (95% CI 0.0–12.9) than patients with undetectable ctDNA alterations (14.5 months (95% CI 3.4–25.6), *p* = 0.028), irrespective of whether they had detectable ctDNA in their pretreatment sample (Figure 3C).

### 2.5. Longitudinal Monitoring of ctDNA SNVs

To explore whether the dynamics of ctDNA SNVs were representative of the treatment responses in the patients during ALK TKI treatment, longitudinal monitoring of NGS-identified SNVs was conducted using ddPCR in six patients. Since ddPCR is limited in its capability to quantify *ALK* rearrangements, we chose to examine patients that had detectable *ALK* mutations in their ctDNA identified by NGS. Furthermore, we also chose patients with other known off-target tumor-associated SNVs, as not all patients acquire *ALK* mutations during treatment. All ddPCR data are available in Table S3.

**Figure 4:**
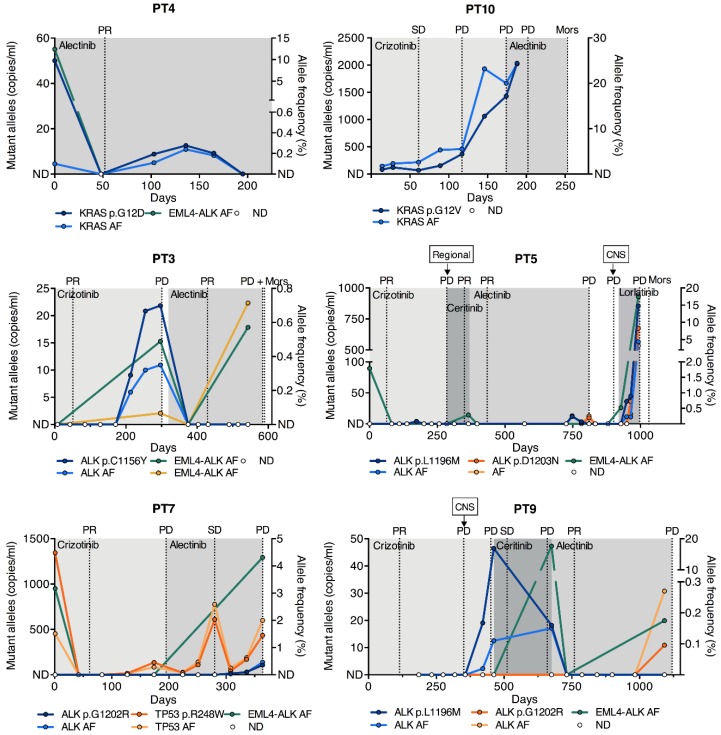
Longitudinal measurements of SNVs. Longitudinal monitoring of SNVs in six patients with ALK, KRAS, and TP53 mutations by ddPCR during ALK TKI treatment. The *x*-axis indicates the time from the start of the treatment in days. The left *y*-axis indicates the mutant allele concentration of the SNV in copies/mL plasma, and the right *y*-axis shows the AF in percent. EML4-ALK rearrangement AFs from NGS analysis are plotted as well. Representative CT scan evaluations are indicated by gridlines, and ALK TKI treatments by shaded areas. PR, partial response; SD, stable disease; Mors, deceased; AF, allele frequency; ND, not detected; CNS, central nervous system; ddPCR, droplet digital PCR.

For all patients achieving partial response (PR) at their first evaluation following ALK TKI initiation, a total clearance of the ctDNA mutant allele concentration in the first blood sample following ALK TKI initiation was observed (PT3, PT4, PT5, PT7, PT9) (Figure 4). This suggested a correlation between tumor response and decreasing mutant allele concentration. Coherently, the mutant allele concentration increased in most cases, leading up to progressive disease (PD), where PD was not determined on the basis of central nervous system (CNS) progression. The increase was detected in the sample prior to the determination of PD by the Response Evaluation Criteria in Solid Tumors (RECIST) and in some cases even earlier. The median lead time of PD detection in ctDNA was 69 days (range 30–113).

Longitudinal monitoring gave significant insight into the clonal tumor evolution of individual patients during treatment. Two of the patients developed *ALK* resistance mutations during crizotinib treatment and were, subsequently, treated with alectinib (PT3 and PT9). The patients achieved PR, and the *ALK* mutant allele was cleared from the circulation and remained undetectable until the last collected sample, approximately one month prior to PD (Figure 4). NGS analysis of the first sample following alectinib initiation revealed that all somatic alterations were cleared for both patients; however, in the last sample collected, the native *ALK* rearrangements were rediscovered (Figure 4). In PT9, the *ALK* p.G1202R mutation, known to confer resistance to every ALK TKI with the exception of lorlatinib [3,5], emerged in connection with the rearrangement. The mechanism of resistance in PT3 remained undetected and may thus be ALK-independent. Another patient acquired an *ALK* p.L1196M mutation after multiple lines of ALK TKIs (PT5). The patient then started lorlatinib treatment, which has demonstrated preclinical activity against this mutation [3]. Nonetheless, the *ALK* mutant allele concentration dramatically increased during lorlatinib treatment, and NGS analysis revealed the emergence of a concurrent *ALK* p.D1203N mutation as early as in the first sample following lorlatinib (Figure 4).

In PT7, the dynamics of a *TP53* p.R248W mutation present at diagnosis followed the clinical response evaluation throughout crizotinib and subsequent alectinib treatment (Figure 4). During alectinib treatment, the *ALK* p.G1202R mutation emerged in unison with the *TP53* p.R248W mutation, however at a lower concentration.

In PT4, the *KRAS* p.G12D mutant allele, which was initially cleared, re-emerged at low levels during the treatment course, even though the patient did not experience PD during the study period (Figure 4). In PT10, who harbored a *KRAS* p.G12V mutation, the mutant allele concentration remained detectable during crizotinib therapy and until PD, although the patient initially achieved stable disease (SD). (Figure 4). This may be the consequence of monitoring a off-target genomic alteration.

## 3. Discussion

The success of targeted therapy is highly dependent on molecular analyses that can inform on the evolution of the genomic composition of a tumor. Since tumor biopsies are not always obtainable or of sufficient quality, the noninvasive analysis of ctDNA is becoming a promising alternative. In this prospective study, we demonstrated the value of ctDNA analyses in NSCLC patients harboring *ALK* rearrangements in their tumor. Using a highly sensitive, commercial CAPP-Seq assay, we demonstrated that ctDNA analyses prior to and early following ALK-TKI treatment initiation may predict clinical outcome and that longitudinal levels of tumor-derived SNVs in the plasma follow clinical response patterns.

We demonstrated a significant association between detectable ctDNA prior to ALK TKI treatment start and inferior PFS irrespective of the TKI administered. The same significant association was found when solely analyzing ctDNA in samples prior to alectinib treatment. Furthermore, we also demonstrated that the presence of ctDNA shortly after treatment initiation correlated with inferior PFS. To the best of our knowledge, the current study is the first to show the possible predictive ability of a comprehensive ctDNA analysis in an ALK-positive NSCLC cohort. Studies conducted with EGFR-mutated patients receiving targeted therapy have reported similar results, as have studies with patients with non-actionable mutations and patients receiving immunotherapy [20,21,22,23]. These studies validate our findings and suggest that ctDNA is generally applicable as a predictive biomarker in lung cancer.

Our descriptive findings of the correlation between longitudinal ctDNA mutant allele concentrations and treatment response are in agreement with previous studies that mostly investigated EGFR-mutated NSCLC patients [14,15,24,25], while only few studies have been performed in ALK-positive patients [26,27,28]. In our study, we assessed not only the *ALK* rearrangement driving the cancer but also other somatic aberrations. We chose to use ddPCR for longitudinal monitoring, as it is extremely sensitive, inexpensive, and rapid compared to NGS analysis. Yet, ddPCR is limited in its capability to quantify structural variants, as a wide variety of genomic breakpoints exist. Nonetheless, we suggest that it may be feasible to track SNVs that exist concurrently with the *ALK* rearrangement for evaluating the response during therapy. For all patients with PR at their first imaging evaluation after treatment start, the mutant allele became undetectable in the first blood sample, further reflecting the utility of ctDNA analysis as a surrogate measure of response. Importantly, longitudinal ctDNA analysis allowed the detection of progression up to months before clinical disease progression was determined, with a median lead time of 69 days. This is in agreement with studies conducted with EGFR-positive patients [14,15] and highlights that the ctDNA level likely acts as a surrogate measure for the overall tumor burden [18,22]. Because tumor burden estimates are standard measures for the evaluation of treatment efficacy, a combination of this together with ctDNA analysis could improve the interpretation of imaging. This is particularly relevant for metastatic patients with multiple tumors, for which RECIST-based tumor burden estimates can be inaccurate.

We detected *ALK* mutations in ctDNA from four patients, and all of these mutations have been previously reported as causing resistance to ALK TKI treatment [3,4,6]. The *ALK* mutations p.L1196M and p.C1156Y were both detected during crizotinib treatment but became undetectable as a result of alectinib treatment, which has been shown to have clinical activity against these mutations [29]. Interestingly, the mutations did not reappear leading up to alectinib-associated progression, indicating that they existed in sub-clones, which were completely eliminated by alectinib treatment. This demonstrates the ability of ctDNA analysis to function as a tool for evaluating clonal evolution.

*ALK* rearrangements have generally been reported to be mutually exclusive with other driver alterations in NSCLC such as *EGFR* and *KRAS* mutations, although few studies have shown that they can coexist [30,31,32]. We found *KRAS* mutations in three of the examined patients, which likely represented different tumor clones, indicative of the heterogenous nature of lung cancer. One patient harbored a *KRAS* p.G12D mutation in the initial treatment-naïve blood sample (PT4) concurrent with an *ALK* rearrangement. The *KRAS* mutation was not detected in the patient’s peripheral blood cells (PBCs), making it unlikely to be due to clonal hematopoiesis. In another patient (PT10), we detected a *KRAS* p.G12V mutation in the first sample following crizotinib, but unfortunately, the pretreatment sample was not available. However, the *KRAS* mutation was found at an AF of 79% in the diagnostic biopsy taken 5 years earlier in connection with curative-intended stereotactic treatment (Table S3). Thus, *KRAS* mutations and *ALK* rearrangements can coexist, and, interestingly, the three patients with this genomic profile had response or stable disease at their first evaluation following ALK TKI start [32].

We identified genomic alterations in the majority of patients (17 out of 24), though *ALK* rearrangements were only identified in plasma samples from 9 patients (39%). This is lower than what was found by other studies with ALK-positive patients [26,27,28]. However, recent studies have elucidated the discrepancies in the detection of SNVs and rearrangements in plasma, comparing different commercial NGS assays [33,34]. Moreover, the capture of structural variants, such as rearrangements, has been reported to be notoriously complex, and a limitation of the CAPP-Seq technology is the potentially inefficient capture of rearranged DNA [18]. Biological factors, such as the number and location of metastatic sites, in individual patients can influence the release of ctDNA to the blood as well [35]. In our study, three out of the seven patients with undetectable ctDNA either did not have metastatic disease or simply had intrathoracic disease. Thus, the detection of ctDNA in the blood is influenced not just by technical limitations but also by features inherent to the tumor and host.

As is often the case when studying ALK-positive patients, the current study is limited by the number of included patients, which mainly renders it hypothesis-generating [26,27,28]. Thus, our results should be validated in a larger cohort of ALK-positive patients. Furthermore, our cohort was slightly heterogenous, since many different ALK TKIs were administered, although most patients received alectinib. Nevertheless, as these patients were not part of a clinical trial, this is a reflection of real-world treatment trajectories for ALK-positive patients. Most of the ctDNA alterations we identified were present at low AFs, and their overall importance for the cancer can thus be questioned. However, we demonstrated that the dynamics of SNVs with low frequencies appeared to follow the clinical response, and patients treated according to ctDNA analyses have been demonstrated to exhibit clinical response to treatment, despite low AFs [35]. Thus, the choice of the detection method is important, as less sensitive methods would not have detected the majority of the mutations reported here.

## 4. Materials and Methods

### 4.1. Patients

Patients were prospectively enrolled in the study at the Department of Oncology at Aarhus University Hospital and Aalborg University Hospital, Denmark, between December 2015 and November 2018. Two patients had additional samples collected previously in another study [36]. Patients were eligible for enrollment if they were above 18 years of age and had histologically proven, ALK-positive non-squamous NSCLC. Patients were included at any time during treatment. Testing for ALK rearrangements was performed as part of the routine diagnostic work-up, using either immunohistochemistry for screening and fluorescence in situ hybridization (ZytoLight ALK Dual Color Break Apart, Zytovision GmBH, Bremerhaven, Germany) for verification in equivocal cases or NGS with the CE-IVD approved Oncomine Solid Tumor DNA and Fusion Transcripts kit (Life Technologies, Carlsbad, CA, USA). Patients were followed from the day of inclusion and during all subsequent lines of treatment. Follow-up was performed using computed tomography (CT) scans every 12 weeks, and response was evaluated according to the Response Evaluation Criteria in Solid Tumors (RECIST) v1.1 [37]. All patients gave informed written consent in accordance with the Declaration of Helsinki. The study was approved by the Central Denmark Region Committees on Biomedical Research Ethics (no. 1-10-72-266-15).

### 4.2. Sampling and Cell-Free DNA Extraction

Peripheral blood samples were collected in connection with routine blood sampling prior to any treatment and approximately every 4th week. We collected 2 × 10 mL of blood in K2EDTA tubes (BD vacutainer^®^, Becton, Dickinson and Company, Franklin Lakes, NJ, USA), and plasma was isolated within two hours by centrifugation (1400× *g* for 10 min). Subsequently, the samples were stored at −80 °C. CfDNA was extracted from 2–4 mL plasma using the cobas^®^ cfDNA Sample Preparation Kit (Roche, Basel, Switzerland) according to the manufacturer’s instructions and eluted in 85 µL elution buffer. DNA from PBCs was extracted using the QIAamp DNA Blood Mini Kit (Qiagen, Hilden, Germany) according to the manufacturer’s protocol. DNA from the diagnostic tissue biopsy was extracted with the QIA Symphony SDP Mini Kit (Qiagen) according to the manufacturer’s instructions.

### 4.3. Next-Generation Sequencing

CfDNA was quantified using the Qubit dsDNA High Sensitivity Kit (Life Technologies). The median DNA input for library preparation was 50 ng. Libraries were prepared with the AVENIO ctDNA Expanded Kit (Roche Sequencing Solutions, Pleasanton, CA, USA) according to the manufacturer’s instructions. Multiplex libraries consisting of 10 unique samples were sequenced on a NextSeq 500 High Output lane (Illumina, San Diego, CA, USA) using 150 bp paired-end runs (median unique depth 4524×, range 1420–9664). Sequencing data were processed using the AVENIO ctDNA Analysis Software version 1.1 with the Expanded Panel Workflow (Roche). Variants were accepted if they were previously reported to the Catalogue of Somatic Mutations in Cancer (COSMIC) or the Cancer Genome Atlas (TCGA). Variants with a mutant AF > 0.1% in any of the Exome Aggregation Consortium (ExAC), 1000 Genomes Project, and Single Nucleotide Polymorphism (dbSNP) databases were excluded. To ensure specificity, variants were required to be present in 3 unique reads and have AF > 0.1% to be accepted [38].

### 4.4. Droplet Digital PCR

DdPCR was performed using the QX200™ AutoDG™ Droplet Digital™ PCR System (Bio-Rad, Hercules, CA, USA). The reaction volume of 22 µL consisted of 2× Supermix for probes (no UTP), 900 nM primers, 250 nM probes, and 9 or 10 µL of cfDNA. All reagents were purchased from Bio-Rad, and assays were either purchased from Bio-Rad or custom-designed by Applied Biosystems (Table S4). All samples were measured as triplicates as a minimum. Wet-lab validated assays were used when possible, and the remaining assays were designed by Bio-Rad or Applied Biosystems and validated in the lab. Data were analyzed using QuantaSoft v.1.7.4.0917 software (Bio-Rad). Each run contained positive and negative controls. Gene Strands (Eurofins Genomics, Ebersberg, Germany) diluted in cfDNA from anonymous blood donors were used as mutation-positive controls. The limit of detection for each assay was determined using blood samples from anonymous donors collected from the blood bank at Aarhus University Hospital, as previously described (Table S4) [39,40]. All mutations chosen for ddPCR analysis were tested in a corresponding PBC sample to rule out clonal hematopoiesis. Results are depicted as both mutant allele concentration and mutant AF, as we have recently shown that the biological variation of ctDNA may affect these two metrics differently [41,42].

### 4.5. Statistics

Patients were dichotomized according to whether somatic alterations in ctDNA were detectable (ctDNA+) or undetectable (ctDNA−) at different time points. Survival analyses were performed by the Kaplan–Meier method, and differences between the groups were determined by the log-rank test. PFS was defined as time from treatment start until PD, death of any cause, or treatment cessation. Patients still undergoing treatment at the time of analysis were censored (22 March 2019). PD was defined as either radiological progression according to RECIST v1.1 criteria or clinical progression. Median follow-up time was estimated by the inverse Kaplan–Meier method. All *p*-values were considered statistically significant if *p* < 0.05. Data analyses were performed using SPSS version 25.0 (IBM, Chicago, IL, USA) and GraphPad Prism 6.0 (GraphPad Software, San Diego, CA, USA).

## 5. Conclusions

In conclusion, we demonstrated that the presence of ctDNA before treatment initiation is associated with inferior PFS, as is the presence of ctDNA shortly after the start of ALK TKI treatment. Interestingly, increased ctDNA levels were identified, leading up to clinical progression, highlighting the potential of ctDNA for real-time monitoring. Our results show that genomic profiling using ctDNA can be of value during all stages of ALK TKI treatment of NSCLC patients. These encouraging findings should be confirmed in prospective, randomized studies to move ctDNA analysis into a clinical setting, which, hopefully, will translate into improved outcomes for cancer patients.

## Figures and Tables

**Table 1:** Patient characteristics at diagnosis (*n* = 24).

Patient Characteristics	
Age, years	
Median (range)	58 (34–84)
Gender	*n* (%)
Female	13 (54)
Male	11 (46)
Smoking status	
Never	8 (33)
Former	12 (50)
Current	3 (13)
No data	1 (4)
Stage	
III	4 (17)
IV	19 (79)
No data	1 (4)
Histology	
Adenocarcinoma	22 (92)
NOS	1 (4)
No data	1 (4)
Prior treatment regimens at study inclusion	
0	14 (58)
1	7 (29)
≥2	3 (13)
ALK TKI regimens during study period	
1	10 (42)
2	13 (54)
4	1 (4)

Abbreviations: NOS, not otherwise specified; ALK, Anaplastic Lymphoma Kinase; TKI, tyrosine kinase inhibitor.

## Supplementary Materials

The following are available online at https://www.mdpi.com/2072-6694/12/4/947/s1, Table S1: Individual patient profiles, Table S2: NGS ctDNA profiles, Table S3: ddPCR ctDNA quantification, Table S4: ddPCR assay information.

## References

[1] Planchard D., Popat S., Kerr K., Novello S., Smit E.F., Faivre-Finn C., Mok T.S., Reck M., Van Schil P.E., Hellmann M.D. (2018). Metastatic non-small cell lung cancer: ESMO Clinical Practice Guidelines for diagnosis, treatment and follow-up. Ann. Oncol..

[2] Ettinger D.S., Aisner D.L., Wood D.E., Akerley W., Bauman J., Chang J.Y., Chirieac L.R., D’Amico T.A., Dilling T.J., Dobelbower M. (2018). NCCN Guidelines Insights: Non–Small Cell Lung Cancer, Version 5.2018. J. Natl. Compr. Cancer Netw..

[3] Gainor J.F., Dardaei L., Yoda S., Friboulet L., Leshchiner I., Katayama R., Dagogo-Jack I., Gadgeel S., Schultz K., Singh M. (2016). Molecular mechanisms of resistance to first- and second-generation ALK inhibitors in ALK -rearranged lung cancer. Cancer Discov..

[4] McCoach C.E., Blakely C.M., Banks K.C., Levy B., Chue B.M., Raymond V.M., Le A.T., Lee C.E., Diaz J., Waqar S.N. (2018). Clinical utility of cell-free DNA for the detection of ALK fusions and genomic mechanisms of ALK inhibitor resistance in non–small cell lung cancer. Clin. Cancer Res..

[5] Shaw A.T., Solomon B.J., Besse B., Bauer T.M., Lin C.-C., Soo R.A., Riely G.J., Ou S.-H.I., Clancy J.S., Li S. (2019). ALK Resistance Mutations and Efficacy of Lorlatinib in Advanced Anaplastic Lymphoma Kinase-Positive Non–Small-Cell Lung Cancer. J. Clin. Oncol..

[6] Jamme P., Descarpentries C., Gervais R., Dansin E., Wislez M., Grégoire V., Richard N., Baldacci S., Rabbe N., Kyheng M. (2019). Relevance of Detection of Mechanisms of Resistance to ALK Inhibitors in ALK-Rearranged NSCLC in Routine Practice. Clin. Lung Cancer.

[7] Katayama R., Shaw A.T., Khan T.M., Mino-Kenudson M., Solomon B.J., Halmos B., Jessop N.A., Wain J.C., Yeo A.T., Benes C. (2012). Mechanisms of acquired crizotinib resistance in ALK-rearranged lung Cancers. Sci. Transl. Med..

[8] Sholl L.M., Aisner D.L., Varella-Garcia M., Berry L.D., Dias-Santagata D., Wistuba I.I., Chen H., Fujimoto J., Kugler K., Franklin W.A. (2015). Multi-institutional Oncogenic Driver Mutation Analysis in Lung Adenocarcinoma: The Lung Cancer Mutation Consortium Experience. J. Thorac. Oncol..

[9] Bosc C., Ferretti G.R., Cadranel J., Audigier-Valette C., Besse B., Barlesi F., Decroisette C., Lantuejoul S., Arbib F., Moro-Sibilot D. (2015). Rebiopsy during disease progression in patients treated by TKI for oncogene-addicted NSCLC. Target Oncol..

[10] Chouaid C., Dujon C., Do P., Monnet I., Madroszyk A., Le Caer H., Auliac J.B., Berard H., Thomas P., Lena H. (2014). Feasibility and clinical impact of re-biopsy in advanced non small-cell lung cancer: A prospective multicenter study in a real-world setting (GFPC study 12-01). Lung Cancer.

[11] Abbosh C., Birkbak N.J., Wilson G.A., Jamal-Hanjani M., Constantin T., Salari R., Le Quesne J., Moore D.A., Veeriah S., Rosenthal R. (2017). Phylogenetic ctDNA analysis depicts early-stage lung cancer evolution. Nature.

[12] Jamal-Hanjani M., Wilson G.A., McGranahan N., Birkbak N.J., Watkins T.B.K., Veeriah S., Shafi S., Johnson D.H., Mitter R., Rosenthal R. (2017). Tracking the Evolution of Non–Small-Cell Lung Cancer. N. Engl. J. Med..

[13] Diehl F., Schmidt K., Choti M.A., Romans K., Goodman S., Li M., Thornton K., Agrawal N., Sokoll L., Szabo S.A. (2008). Circulating mutant DNA to assess tumor dynamics. Nat. Med..

[14] Sorensen B.S., Wu L., Wei W., Tsai J., Weber B., Nexo E., Meldgaard P. (2014). Monitoring of epidermal growth factor receptor tyrosine kinase inhibitor-sensitizing and resistance mutations in the plasma DNA of patients with advanced non-small cell lung cancer during treatment with erlotinib. Cancer.

[15] Oxnard G.R., Paweletz C.P., Kuang Y., Mach S.L., O’Connell A., Messineo M.M., Luke J.J., Butaney M., Kirschmeier P., Jackman D.M. (2014). Noninvasive detection of response and resistance in EGFR-mutant lung cancer using quantitative next-generation genotyping of cell-free plasma DNA. Clin. Cancer Res..

[16] Murtaza M., Dawson S.-J.J., Pogrebniak K., Rueda O.M., Provenzano E., Grant J., Chin S.-F.F., Tsui D.W.Y.Y., Marass F., Gale D. (2015). Multifocal clonal evolution characterized using circulating tumour DNA in a case of metastatic breast cancer. Nat. Commun..

[17] Murtaza M., Dawson S.J., Tsui D.W.Y., Gale D., Forshew T., Piskorz A.M., Parkinson C., Chin S.F., Kingsbury Z., Wong A.S.C. (2013). Non-invasive analysis of acquired resistance to cancer therapy by sequencing of plasma DNA. Nature.

[18] Newman A.M., Bratman S.V., To J., Wynne J.F., Eclov N.C.W., Modlin L.A., Liu C.L., Neal J.W., Wakelee H.A., Merritt R.E. (2014). An ultrasensitive method for quantitating circulating tumor DNA with broad patient coverage. Nat. Med..

[19] Newman A.M., Lovejoy A.F., Klass D.M., Kurtz D.M., Chabon J.J., Scherer F., Stehr H., Liu C.L., Bratman S.V., Say C. (2016). Integrated digital error suppression for improved detection of circulating tumor DNA. Nat. Biotechnol..

[20] Mok T., Wu Y.-L.L., Lee J.S., Yu C.-J.J., Sriuranpong V., Sandoval-Tan J., Ladrera G., Thongprasert S., Srimuninnimit V., Liao M. (2015). Detection and dynamic changes of EGFR mutations from circulating tumor DNA as a predictor of survival outcomes in NSCLC Patients treated with first-line intercalated erlotinib and chemotherapy. Clin. Cancer Res..

[21] Wang Z., Cheng Y., An T., Gao H., Wang K., Zhou Q., Hu Y., Song Y., Ding C., Peng F. (2018). Detection of EGFR mutations in plasma circulating tumour DNA as a selection criterion for first-line gefitinib treatment in patients with advanced lung adenocarcinoma (BENEFIT): A phase 2, single-arm, multicentre clinical trial. Lancet Respir. Med..

[22] Winther-Larsen A., Demuth C., Fledelius J., Madsen A.T., Hjorthaug K., Meldgaard P., Sorensen B.S. (2017). Correlation between circulating mutant DNA and metabolic tumour burden in advanced non-small cell lung cancer patients. Br. J. Cancer.

[23] Goldberg S.B., Narayan A., Kole A.J., Decker R.H., Teysir J., Carriero N.J., Lee A., Nemati R., Nath S.K., Mane S.M. (2018). Early Assessment of Lung Cancer Immunotherapy Response via Circulating Tumor DNA. Clin. Cancer Res..

[24] Sacher A.G., Paweletz C., Dahlberg S.E., Alden R.S., O’Connell A., Feeney N., Mach S.L., Jänne P.A., Oxnard G.R. (2016). Prospective Validation of Rapid Plasma Genotyping for the Detection of *EGFR* and *KRAS* Mutations in Advanced Lung Cancer. JAMA Oncol..

[25] Marchetti A., Palma J.F., Felicioni L., De Pas T.M., Chiari R., Del Grammastro M., Filice G., Ludovini V., Brandes A.A., Chella A. (2015). Early Prediction of Response to Tyrosine Kinase Inhibitors by Quantification of EGFR Mutations in Plasma of NSCLC Patients. J. Thorac. Oncol..

[26] Dagogo-Jack I., Brannon A.R., Ferris L.A., Campbell C.D., Lin J.J., Schultz K.R., Ackil J., Stevens S., Dardaei L., Yoda S. (2018). Tracking the Evolution of Resistance to ALK Tyrosine Kinase Inhibitors Through Longitudinal Analysis of Circulating Tumor DNA. JCO Precis. Oncol..

[27] Wang Y., Tian P.-W., Wang W.-Y., Wang K., Zhang Z., Chen B.-J., He Y.-Q., Li L., Liu H., Chuai S. (2016). Noninvasive genotyping and monitoring of anaplastic lymphoma kinase (ALK) rearranged non-small cell lung cancer by capture-based next-generation sequencing. Oncotarget.

[28] Horn L., Whisenant J.G., Wakelee H., Reckamp K.L., Qiao H., Leal T.A., Du L., Hernandez J., Huang V., Blumenschein G.R. (2019). Monitoring Therapeutic Response and Resistance: Analysis of Circulating Tumor DNA in Patients With ALK+ Lung Cancer. J. Thorac. Oncol..

[29] Sakamoto H., Tsukaguchi T., Hiroshima S., Kodama T., Kobayashi T., Fukami T.A., Oikawa N., Tsukuda T., Ishii N., Aoki Y. (2011). CH5424802, a selective ALK inhibitor capable of blocking the resistant gatekeeper mutant. Cancer Cell.

[30] Gainor J.F., Varghese A.M., Ou S.-H.I., Kabraji S., Awad M.M., Katayama R., Pawlak A., Mino-Kenudson M., Yeap B.Y., Riely G.J. (2013). ALK rearrangements are mutually exclusive with mutations in EGFR or KRAS: An analysis of 1,683 patients with non-small cell lung cancer. Clin. Cancer Res..

[31] Ulivi P., Chiadini E., Dazzi C., Dubini A., Costantini M., Medri L., Puccetti M., Capelli L., Calistri D., Verlicchi A. (2016). Nonsquamous, Non-Small-Cell Lung Cancer Patients Who Carry a Double Mutation of EGFR, EML4-ALK or KRAS: Frequency, Clinical-Pathological Characteristics, and Response to Therapy. Clin. Lung Cancer.

[32] Schmid S., Gautschi O., Rothschild S., Mark M., Froesch P., Klingbiel D., Reichegger H., Jochum W., Diebold J., Früh M. (2017). Clinical Outcome of ALK -Positive Non–Small Cell Lung Cancer (NSCLC) Patients with De Novo EGFR or KRAS Co-Mutations Receiving Tyrosine Kinase Inhibitors (TKIs). J. Thorac. Oncol..

[33] Supplee J.G., Milan M.S.D., Lim L.P., Potts K.T., Sholl L.M., Oxnard G.R., Paweletz C.P. (2019). Sensitivity of next-generation sequencing assays detecting oncogenic fusions in plasma cell-free DNA. Lung Cancer.

[34] Stetson D., Ahmed A., Xu X., Nuttall B.R.B., Lubinski T.J., Johnson J.H., Barrett J.C., Dougherty B.A. (2019). Orthogonal Comparison of Four Plasma NGS Tests With Tumor Suggests Technical Factors are a Major Source of Assay Discordance. JCO Precis. Oncol..

[35] Aggarwal C., Thompson J.C., Black T.A., Katz S.I., Fan R., Yee S.S., Chien A.L., Evans T.L., Bauml J.M., Alley E.W. (2019). Clinical Implications of Plasma-Based Genotyping With the Delivery of Personalized Therapy in Metastatic Non–Small Cell Lung Cancer. JAMA Oncol..

[36] Sandfeld-Paulsen B., Folkersen B.H., Rasmussen T.R., Meldgaard P., Sorensen B.S. (2016). Gene expression of the egf system—a prognostic model in non–small cell lung cancer patients without activating EGFR mutations. Transl. Oncol..

[37] Eisenhauer E.A., Therasse P., Bogaerts J., Schwartz L.H., Sargent D., Ford R., Dancey J., Arbuck S., Gwyther S., Mooney M. (2009). New response evaluation criteria in solid tumours: Revised RECIST guideline (version 1.1). Eur. J. Cancer.

[38] Volckmar A.L., Sültmann H., Riediger A., Fioretos T., Schirmacher P., Endris V., Stenzinger A., Dietz S. (2018). A field guide for cancer diagnostics using cell-free DNA: From principles to practice and clinical applications. Genes Chromosom. Cancer.

[39] Demuth C., Winther-Larsen A., Madsen A.T., Meldgaard P., Sorensen B.S. (2018). A method for treatment monitoring using circulating tumour DNA in cancer patients without targetable mutations. Oncotarget.

[40] Milbury C.A., Zhong Q., Lin J., Williams M., Olson J., Link D.R., Hutchison B. (2014). Determining lower limits of detection of digital PCR assays for cancer-related gene mutations. Biomol. Detect. Quantif..

[41] Hojbjerg J.A., Madsen A.T., Schmidt H.H., Sorensen S.F., Stougaard M., Meldgaard P., Sorensen B.S. (2019). Intra-individual variation of circulating tumour DNA in lung cancer patients. Mol. Oncol..

[42] Madsen A.T., Hojbjerg J.A., Sorensen B.S., Winther-Larsen A. (2019). Day-to-day and within-day biological variation of cell-free DNA. EBioMedicine.

